# Anti-inflammatory Effects of Alcohol Are Associated with JNK-STAT3 Downregulation in an *In Vitro* Inflammation Model in HepG2 Cells

**DOI:** 10.1155/2021/6622701

**Published:** 2021-03-18

**Authors:** Katharina Mörs, Ramona Sturm, Jason-Alexander Hörauf, Shinwan Kany, Paola Cavalli, Jazan Omari, Maciej Powerski, Alexey Surov, Ingo Marzi, Aleksander J. Nowak, Borna Relja

**Affiliations:** ^1^Department of Trauma, Hand and Reconstructive Surgery, University Hospital Frankfurt, Goethe University, Frankfurt am Main, Germany; ^2^Department of Cardiology with Emphasis on Electrophysiology, University Heart Centre, University Hospital Hamburg-Eppendorf, 20251 Hamburg, Germany; ^3^Experimental Radiology, Department of Radiology and Nuclear Medicine, Otto von Guericke University, Magdeburg, Germany

## Abstract

**Background:**

In several preclinical and *in vitro* models of acute inflammation, alcohol (ethanol, EtOH) has been described as an immunomodulatory agent. Similarly, in different pathologies, clinical observations have confirmed either pro- or anti-inflammatory effects of EtOH. The liver plays an important role in immunity and alcohol metabolism; therefore, we analysed dose- and time-dependent effects of EtOH on the inflammatory response of human liver cells in an *in vitro* model of acute inflammation.

**Methods:**

HepG2 cells were stimulated with IL-1*β* and subsequently exposed to EtOH in a low or high dose (85 mM, LoD or 170 mM, HiD) for 1 h (acute exposure) or 72 h (prolonged exposure). IL-6 and TNF-*α* release was determined by ELISA. Cell viability, adhesion of isolated neutrophils to HepG2 monolayers, their ICAM-1 expression, and the activation of stress-induced protein kinase/c-Jun N-terminal kinase (SAPK/JNK) or signal transducer and activator of transcription 3 (STAT3) were analysed.

**Results:**

In this experimental design, EtOH did not markedly change the cell viability. Acute and prolonged exposure to EtOH significantly reduced dose-independent IL-1*β*-induced IL-6 and TNF-*α* release, as well as adhesion capacity to pretreated HepG2 cells. Acute exposure to EtOH significantly decreased the percentage of ICAM-1-expressing cells. IL-1*β* stimulation notably increased the activation of SAPK/JNK. However, low-dose EtOH exposure reduced this activation considerably, in contradiction to high-dose EtOH exposure. Acute exposure to LoD EtOH significantly diminished the IL-1*β*-induced STAT3 activation, whereas an acute exposure of cells to either HiD EtOH or in a prolonged setting showed no effects on STAT3 activation.

**Conclusion:**

EtOH exerts anti-inflammatory potential in this *in vitro* model of hepatic inflammation. These effects are associated with the reduced activation of JNK/STAT3 by EtOH, particularly in the condition of acute exposure to low-dose EtOH.

## 1. Introduction

Alcohol use is a well-known risk factor for liver diseases, which evokes a local inflammatory response resulting in hepatic tissue remodelling with subsequent liver cirrhosis and thus significantly impaired liver function [1–4]. The liver plays an important role in several major physiological functions, including digestion, coagulation, or regulation of immune response; thus, its dysfunction and end-stage liver disease can lead to high mortality rates [5].

In regard to trauma patients (TP), alcohol use is of significant importance, because of the high prevalence of both chronic alcoholism and acute intoxication in patients suffering from severe injuries [6, 7]. In the study of Nau et al., liver cirrhosis was associated with increased in-hospital mortality rates and organ complications [8]. As liver cirrhosis constitutes the final stage of alcoholic liver disease, the aforementioned findings suggest that chronic alcohol usage evokes an impaired outcome after traumatic injury. Similarly, patients with evidenced chronic alcohol misuse had increased risk of pneumonia and other infections compared to patients without a history of alcohol use [9]. Spies et al. reported significantly prolonged intensive care unit stays in chronic alcoholics after severe injury due to more developed complications, including more frequent pneumonia cases and severe cases of alcohol withdrawal syndrome [10]. In contrast, acute alcohol use in patients suffering from traumatic brain injury (TBI) was associated with lower incidence of admission coagulopathy, less pneumonia rates, and reduced in-hospital mortality compared to not-intoxicated TP [11, 12]. However, Raj et al. have observed a dose-dependent correlation influencing the mortality outcome. They found a reduced mortality rate six months after TBI in patients who had low blood alcohol concentration levels (BAC, <2.3‰) in comparison to TBI patients with no or high BAC (>2.3‰) [13]. In contradiction, in a binge drinking study, acutely intoxicated TP with positive BAC (>0.5‰) did not have significant differences in outcome parameters including pneumonia, sepsis, organ failure, and in-hospital mortality rate compared to controls [14].

Interestingly, in Hadjizacharia et al., the positive BAC level was considered to be a risk factor for higher mortality after traumatic injury [15]. The contradictory results of various clinical study outcomes may lie underneath the still not comprehensively elucidated EtOH impact on the immune system. Thus, to elucidate the possible mechanisms of alcohol immunomodulatory character, several clinical studies involving EtOH intake have reported altered systemic levels of proinflammatory mediators, such as tumor necrosis factor- (TNF-) *α*, interleukins 1 and 6 (IL-1 and IL-6, respectively), or interferon (IFN) [14, 16, 17].

This phenomenon has been observed in both acute alcohol use and traumatic injury animal models. In a rat model of isolated haemorrhagic shock, animals that received a single oral dose of alcohol prior to the experiment exhibited decreased local hepatic and systemic inflammation, as shown by, e.g., decreased IL-6 levels or diminished neutrophil infiltration in the liver [18]. Treating polymorphonuclear neutrophils (PMN), isolated from volunteers' cell-free blood supernatants and purified afterwards, with 0-0.8% EtOH for 1 h after their stimulation with LPS and LPS+IFN-*γ*-stimulation for up to 24 h decreased significantly IL-8 and TNF-*α* production levels by PMN in a concentration-dependent manner [19]. Stimulating human lung epithelial cells with proinflammatory cytokines and subsequently treating them with EtOH significantly diminished IL-8 and IL-6 levels as well as the adhesion rates of neutrophils [20, 21]. Treating human monocytes with EtOH elevated levels of the anti-inflammatory cytokine IL-10 [22].

The underlying mechanisms of alcohol immunomodulatory properties include the molecular involvement of a protein complex known as the nuclear factor kappa-light-chain-enhancer of activated B-cells (NF-*κ*B), an important DNA transcription regulating factor [21, 23, 24]. However, mechanistic response to extracellular stress stimuli involves multiple signaling pathways. A particular example constitutes the stress-activated protein kinase/c-Jun N-terminal kinase (SAPK/JNK), a signaling cascade component from the mitogen-activated protein kinase (MAPK) family that plays a decisive role in regulating gene expression in response to extracellular stressors [25]. The JNK pathway can be stimulated by either environmental stress mediators (e.g., in response to UV radiation or reactive oxygen species) or specific cell signals and cytokines [26]. The signal from proinflammatory and/or proapoptotic cytokines (e.g., IL-6, IL-1*β*, or TNF-*α*) is transduced through other MAPK family constituents and leads to the activation of JNKs by double phosphorylation [27]. Consequently, the activated c-Jun N-terminal kinase double phosphorylates serine or threonine residues on several target transcription factors, including c-Jun protein, signal transducer and activator of transcription 3 protein (STAT3), or p53, which leads to respective responses, in particular inflammation and/or apoptosis [28]. It has been shown that acute alcohol intake can activate the JNK pathway in hepatocytes through the increased oxidative stress levels, caused intracellularly by alcohol and its metabolites [29]. In addition, alcohol-induced activation of JNK is able to enhance the release of proinflammatory cytokines (e.g., TNF-*α*) in monocytes [30], which further suggests its role as an important immune modulator [31].

Furthermore, STAT3 acts as an important downstream signal mediator, and it is involved in cytokine production *via* the MAPK-STAT3 axis upregulation [32, 33]. Samavati et al. reported STAT3 phosphorylation by JNK, resulting in IL-1*β* and IL-6 expression in murine macrophages upon LPS stimulation [34]. Concerning EtOH, Norkina et al. observed increased IL-10 levels to be associated with enhanced activation of STAT3 in the presence of alcohol and/or LPS [22]. Taken together, the observed immunosuppressive effects of alcohol might be exerted *via* several molecular pathways.

In this study, we investigated alcohol dose- as well as time-dependent effects on the inflammatory response of human liver epithelial-like cells *in vitro.* Additionally, we aimed to analyse the potential underlying mechanisms of alcohol properties regarding the MAPK-STAT3 axis in cellular signalling.

## 2. Material and Methods

### 2.1. Cell Culture

Human hepatocellular carcinoma cells HepG2 (Cell Line Services, Heidelberg, Germany) were cultured under standardized conditions at 37°C under 5% CO_2_ in RPMI-1640 medium (Seromed, Berlin, Germany), supplemented with 10% heat-inactivated fetal calf serum (FCS), 100 IU/ml penicillin and 100 *μ*g/ml streptomycin (Gibco, Karlsruhe, Germany), and 20 mM HEPES buffer (Sigma, Steinheim, Germany). Culture media were replaced 2-3x per week.

Peripheral blood mononuclear neutrophils (PMN) were isolated from healthy volunteers by density gradient centrifugation (Polymorphprep, Nycomed, Oslo, Norway) according to the manufacturer's guidelines. After isolation, both the number and viability of PMN were analysed via the trypan blue exclusion assay. Cells with a purity of >95% were applied for experiments, and cells were cultured in RPMI-1640 medium.

### 2.2. Cell Stimulation

**Figure 1:**
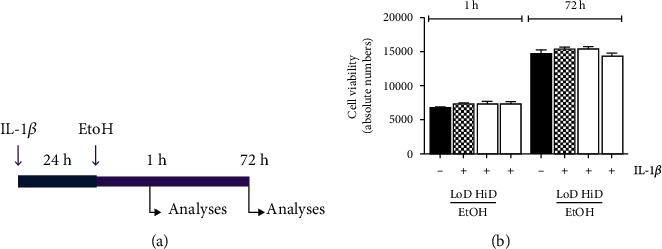
Schematic timeline of the experimental design is shown (a). Absolute HepG2 cell viability after stimulation with IL-1*β* (1 ng/ml) and low (LoD, 85 mM)- and high (HiD, 170 mM)-dose EtOH pretreatment for 1 h (acute) or 72 h (prolonged) is represented (b). Longer exposure to EtOH did not affect cell viability. Unstimulated controls (-); IL-1*β*-stimulated samples (+).

The concentrations of IL-1*β* and EtOH are based on both our own and other groups' previous studies, allowing better comparison of data [20, 21]. HepG2 cells were stimulated with recombinant human IL-1*β* (1 ng/ml, R&D Systems, Wiesbaden, Germany) for 24 h. Afterwards, EtOH was added in two different concentrations at 85 (LoD) and 170 mM (HiD, corresponding to 0.5-1% *v*/*v* EtOH, i.e., 4.0–7.9 mg/ml EtOH) in order to analyse dose dependency. Incubation periods of 1 h (acute) or 72 h (prolonged) were followed in order to study the effects of different duration of alcohol exposure. The schematic timeline of the experimental design is depicted in Figure 1(a).

### 2.3. Measurement of Cell Viability

Cell viability was assessed by using the Calcein^AM^ Cell Viability Assay (R&D Systems, Minneapolis, Minnesota, US). Calcein^AM^ as a nonfluorescent hydrophobic compound easily permeates intact live cells. The hydrolysis of Calcein^AM^ by intracellular esterases produces calcein, which is a hydrophilic strongly fluorescent compound well retaining in the cell cytoplasm. After seeding the cells in clear bottom, black-walled 96-well plates (BD Biosciences, Franklin Lakes, New Jersey, US) and treating according to the stimulation protocol, Calcein^AM^ Working Solution was prepared according to the manufacturer's instructions. Then, the media were removed, the cells were washed twice with Calcein^AM^ DW Buffer (100 *μ*l and 50 *μ*l), and the Calcein^AM^ Working Solution (50 *μ*l) was added to the wells and incubated for 30 min. The fluorescence intensity was detected by a Twinkle LB 970 Microplate Fluorometer (with a 490 nm excitation filter and a 520 nm emission filter, software MikroWin 2000). To read out the data, a standard curve with defined cell numbers was created. Since the number of viable cells is proportional to the fluorescence intensity, the viability of cells was calculated accordingly.

### 2.4. Quantification of Cytokine Production

To determine the effects of EtOH on the cytokine release, HepG2 cells were exposed to either LoD or HiD EtOH for 1 or 72 h after their stimulation with IL-1*β* for 24 h. Then, cell culture supernatants were collected, and IL-6 and TNF-*α* levels were determined using Diaclone IL6/TNF-*α* ELISA Sets (Diaclone, France), according to manufacturer's instructions using the Infinite M200 microplate reader (Tecan, Männedorf, Switzerland).

### 2.5. Monolayer Adhesion Assay

The assay was performed as described previously [20]. To analyse the adhesion capacity of PMN to monolayers of HepG2 after pretreatment, firstly, the HepG2 cells were allocated into RPMI-1640 medium in proper multiplates (24 wells, Sarstedt). After reaching the confluence of ~80%, the IL-1*β* and EtOH stimulation of the culture followed, as described before. The PMN cells were adjusted to 5 × 10^4^ vital cells per well and then cultivated over monolayers of HepG2. The coculture was incubated (37°C, 5% CO_2_) for 15 minutes; then, unattached PMN cells were removed with warmed up RPMI-1640 medium—this step was applied three times. Afterwards, the adherent PMN cells were fixed with 1% glutaraldehyde and analysed under a phase-contrast microscope (×10, 5 × 0.25 mm^2^). The PMN adhesion rate was calculated and expressed in percentage in the ratio to unstimulated PMN control cells.

### 2.6. CD54 Expression

After stimulation with IL-1*β* and following exposure to EtOH as described above, HepG2 cells were washed with PBS supplements, containing 0.5% bovine serum albumin (BSA). Subsequently, cells were incubated with a fluorescein-conjugated monoclonal antibody directed against CD54/intercellular adhesion molecule- (ICAM-) 1 (BBA20; R&D Systems) for 60 min at 4°C as described previously [20, 35]. CD54 expression was analysed by flow cytometry using FACSCalibur (1 × 10^4^ cells/scan were counted; BD Biosciences, Heidelberg, Germany) and expressed as a percentage of positive cells (%). A mouse IgG1 fluorescein antibody (IC002F; R&D Systems) was used as the isotype control.

### 2.7. SAPK/JNK and STAT3

A PathScan® Phospho-SAPK/JNK (Thr183/Tyr185) Sandwich ELISA Kit (Cell Signaling Technology) detects endogenous levels of Phospho-SAPK/JNK protein. The FastScan™ Phospho-Stat3 (Ser727) ELISA Kit is a sandwich enzyme-linked immunosorbent assay (ELISA) that detects endogenous levels of Stat3 when phosphorylated at Ser727 (Cell Signaling Technology). Briefly, all solutions including detection antibodies, HRP-linked antibodies, wash buffers, and cell lysis buffers were prepared as suggested by the manufacturer. Then, cell lysates were prepared as well according to the manufacturer's instructions. After treating the cells as described before, the media were removed, and the cells were washed with ice-cold PBS. Subsequently, the cells were lysed with lysing or extraction buffer as suggested by the manufacturer. After final sonication and microcentrifugation, the supernatant was applied for the arrays as described in each PathScan® Sandwich ELISA Kit. The spectrophotometric determination was applied at the absorbance at 450 nm within 30 min after adding STOP Solution using the Infinite M200 microplate reader (Tecan, Männedorf, Switzerland).

### 2.8. Statistical Analysis

GraphPad Prism 6 (GraphPad Software Inc., San Diego, CA) was used to perform the statistical analyses. Differences between groups were determined by the Kruskal-Wallis test followed by post hoc Dunn's test. A *p* value below 0.05 was considered significant. Data are given as the mean ± standard error of the mean (s.e.m.). All experiments were performed 3-5 times.

## 3. Results

### 3.1. Viability of HepG2 Cells after Stimulation with IL-1*β* and Subsequent EtOH Treatment

Cell viability of HepG2 cells was not markedly altered by IL-1*β* stimulation. Subsequent acute (1 h) exposure of HepG2 cells to EtOH had no effects on cell viability, neither in LoD nor in HiD setting. Similarly, prolonged exposure to EtOH did not alter cellular viability (Figure 1(b)).

### 3.2. Release of IL-6 and TNF-*α* after Stimulation with IL-1*β* and Subsequent EtOH Treatment of HepG2 Cells

In order to analyse the time- and dose-dependent effects of EtOH on the release of proinflammatory cytokines by HepG2 cells, IL-6 and TNF-*α* levels in the supernatants were determined after stimulation with IL-1*β* and subsequent acute or prolonged EtOH exposure.

**Figure 2:**
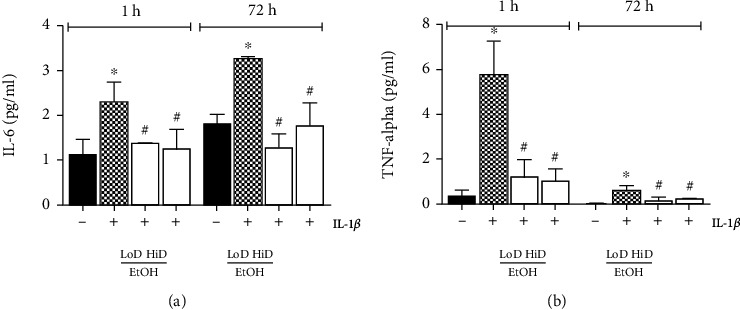
Interleukin- (IL-) 6 (a) and tumor necrosis factor- (TNF-) *α* (b) levels expressed by HepG2 cells were determined in cell-free supernatants. Cells were stimulated with IL-1*β* (1 ng/ml) and treated with low (LoD, 85 mM)- or high (HiD, 170 mM)-dose EtOH for 1 h (acute) or 72 h (prolonged). IL-1*β* stimulation resulted in increased levels of IL-6 and TNF-*α*, while EtOH exposure significantly decreased the cytokine expression. Unstimulated controls (-); IL-1*β*-stimulated samples (+). *p* < 0.05: ^∗^*vs*. unstimulated controls, ^#^*vs*. stimulated and EtOH-untreated controls.

Stimulation with IL-1*β* resulted in significantly increased IL-6 as well as TNF-*α* release in HepG2 cells to 2.29 ± 0.46 and 5.74 ± 1.52 pg/ml compared to sham 1.05 ± 0.41 and 0.36 ± 0.24 pg/ml, respectively (*p* < 0.05, Figure 2). Subsequent acute exposure to EtOH significantly decreased IL-6 as well as TNF-*α* levels. This was observed for LoD (IL-6: 1.37 ± 0.03 and TNF-*α*: 1.19 ± 0.79 pg/ml) as well as for HiD EtOH (IL-6: 1.25 ± 0.43 and TNF-*α*: 1.02 ± 0.55 pg/ml) *vs*. stimulated control (*p* < 0.05, Figure 2).

After prolonged exposure to EtOH either to LoD or to HiD, IL-1*β*-induced IL-6 and TNF-*α* release again decreased significantly in comparison with stimulated controls (LoD, IL-6: 1.27 ± 0.31*vs*. 3.27 ± 0.04 and TNF-*α*: 0.17 ± 0.16*vs*. 0.53 ± 0.18 pg/ml; HiD, IL-6: 1.77 ± 0.51*vs*. 3.27 ± 0.04 and TNF-*α*: 0.24 ± 0.16*vs*. 0.53 ± 0.18 pg/ml, *p* < 0.05, Figure 2).

### 3.3. Neutrophil Adhesiveness to HepG2 Cells after IL-1*β* Stimulation and Subsequent EtOH Exposure

**Figure 3:**
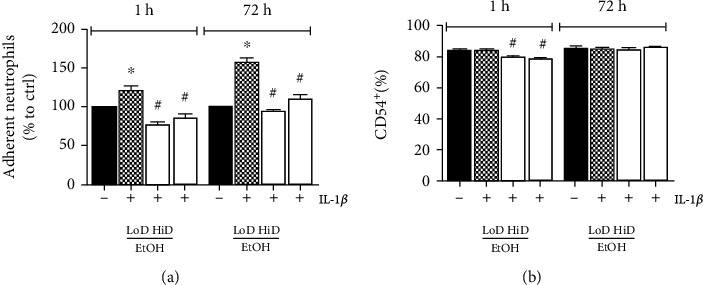
Percentage of attached neutrophils to HepG2 monolayers after stimulation with IL-1*β* (1 ng/ml) and treatment with low (LoD, 85 mM)- or high (HiD, 170 mM)-dose EtOH for 1 h (acute) or 72 h (prolonged) (a). The expression levels of CD54 in HepG2 cells by percentage are shown (b). Unstimulated controls (-); IL-1*β*-stimulated samples (+). *p* < 0.05: ^∗^*vs*. unstimulated controls, ^#^*vs*. stimulated and EtOH-untreated controls.

Neutrophil adhesion to HepG2 monolayers was significantly enhanced after IL-1*β* stimulation compared to unstimulated controls (121.00 ± 5.96 compared to control 100.00%, *p* < 0.05, Figure 3(a)). Following exposure to LoD as well as HiD EtOH for 1 h significantly decreased neutrophil adhesion capacity compared to stimulated controls (76.53 ± 3.96 LoD EtOH to 85.07 ± 6.01% HiD EtOH, *p* < 0.05, Figure 3(a)), while there was no significant difference between both EtOH concentrations. Also, subacute EtOH treatment for 72 h resulted in significantly diminished adhesiveness of PMN to HepG2 monolayers (91.00 ± 2.51 LoD EtOH to 113.70 ± 9.64% HiD EtOH, *p* < 0.05, Figure 3(a)), again showing no significant differences when comparing both EtOH doses.

### 3.4. CD54 (ICAM-1) Expression in HepG2 Cells upon EtOH Treatment following IL-1*β* Stimulation

We analysed alcohol effects on the expression of CD54 as an intercellular adhesion molecule involved in leukocyte transmigration during states of inflammation. Stimulation with IL-1*β* (1 ng/ml) did not alter the percentage of cells expressing CD54 (84.30 ± 1.26 compared to unstimulated 84.47 ± 1.06%, Figure 3(b)). Acute EtOH significantly reduced the percentage of cells expressing CD54 in LoD (85 mM) as well as HiD (170 mM) when compared to unstimulated and stimulated controls (79.73 ± 1.15 LoD EtOH to 78.92 ± 0.71% HiD EtOH, *p* < 0.05, Figure 3(b)). However, there was no difference between both EtOH concentrations. In contradiction, prolonged 72 h EtOH exposure did not show any significant differences (84.95 ± 1.12 LoD EtOH and 86.48 ± 0.55% HiD EtOH, compared to unstimulated 86.13 ± 1.01 and stimulated 85.03 ± 1.41% control, Figure 3(b)), with no difference between concentrations as well.

### 3.5. SAPK/JNK Activation after Stimulation with IL-1*β* and Subsequent EtOH Treatment of HepG2 Cells

In order to determine SAPK/JNK activation, we analysed phosphorylation of Thr183/Tyr185 after stimulation with IL-1*β* and after subsequent EtOH exposure.

**Figure 4:**
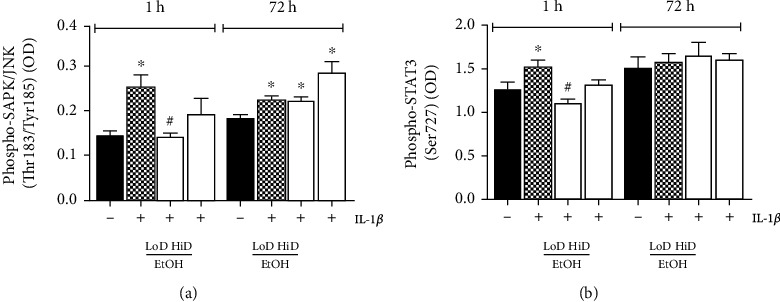
Protein expression levels of phosphorylated SAPK/JNK (Thr183/Tyr185, (a) or phosphorylated STAT3 (Ser727, (b) levels in HepG2 cells are shown. Cells were stimulated with IL-1*β* (1 ng/ml) and treated with low (LoD, 85 mM)- or high (HiD, 170 mM)-dose EtOH for 1 h (acute) or 72 h (prolonged). Unstimulated controls (-); IL-1*β*-stimulated samples (+). *p* < 0.05: ^∗^*vs*. unstimulated controls, ^#^*vs*. stimulated and EtOH-untreated controls.

IL-1*β* stimulation resulted in significantly enhanced SAPK/JNK activation (0.26 ± 0.03 to 0.15 ± 0.01, *p* < 0.05, Figure 4(a)). Subsequent acute LoD and HiD EtOH reduced SAPK/JNK activation when compared to stimulated controls (0.15 ± 0.01 LoD EtOH and 0.20 ± 0.04 HiD EtOH to 0.26 ± 0.03 of the control, *p* < 0.05 in LoD EtOH, Figure 4(a)). Subacute EtOH exposure did not markedly change the SAPK/JNK activation, hinting at dose as well as time dependency of alcohol properties. In prolonged 72 h exposure, a minor phosphorylation increase rate after HiD EtOH treatment was noticed in comparison to LoD EtOH (0.29 ± 0.03 HiD EtOH and 0.22 ± 0.01 LoD EtOH to 0.23 ± 0.01 of the stimulated control, Figure 4(a)), although no significant changes between LoD EtOH prolonged exposure and stimulated control were observed.

### 3.6. STAT3 Activation in HepG2 Cells after Stimulation with IL-1*β* and Subsequent EtOH Exposure

STAT3 phosphorylation at Ser727 was increased upon IL-1*β* stimulation (1.26 ± 0.10 to 1.53 ± 0.07, *p* < 0.05, Figure 4(b)). Exposing HepG2 cells to LoD EtOH (85 mM) for 1 h afterwards resulted in significantly reduced STAT3 activation (1.11 ± 0.05 to 1.53 ± 0.07, *p* < 0.05, Figure 4(b)). Treatment with HiD EtOH (170 mM) for 1 h decreased IL-1*β*-induced STAT3 activation; however, this was not significant compared to IL-1*β*-stimulated controls (1.32 ± 0.05 to 1.53 ± 0.07, Figure 4(b)). Also, subacute (prolonged) EtOH exposure (72 h) in both concentrations had no effect on STAT3 activation (1.65 ± 0.16 LoD EtOH and 1.60 ± 0.08 HiD EtOH to 1.57 ± 0.10 stimulated control, Figure 4(b)).

## 4. Discussion

In this *in vitro* study, we analysed the immunomodulatory effects of either acute or prolonged exposure of human liver cells to alcohol after their IL-1*β*-induced inflammatory response. In order to determine dose dependency, two different alcohol concentrations were applied. The choice of active alcohol concentrations and time of the incubation was based on our own previous experimental works [20, 35] and other group's experiences [19] and in consonance with already established models in alcohol-focused studies [36]. The concentration of alcohol in our approach corresponded to 0.5-1% *v*/*v* of total EtOH content in the media, which is still tolerable by most of the cultivated cells, malignant and primary alike [37]. Both acute and prolonged exposure to each concentration of alcohol significantly decreased the release of the proinflammatory cytokines IL-6 and TNF-*α* upon the cellular stimulation with IL-1*β*. Similarly, adhesiveness of isolated neutrophils to stimulated HepG2 cells was significantly diminished in each experimental setting. In addition, we have stimulated HepG2 cells with IL-6 (10 ng/ml) for 24 hours and performed further evaluations of IL-8 in the supernatants and functional assay. In line with the data provided in the present manuscript, upon IL-1*β* stimulation, both acute and prolonged exposure to each concentration of alcohol decreased the release of the proinflammatory cytokine IL-8, and adhesiveness of isolated neutrophils to IL-6-stimulated HepG2 cells was significantly diminished in each experimental setting (data not shown). Additionally, we observed a significantly increased phosphorylation rate of SAPK/JNK after IL-1*β* stimulation and significant reduction of the SAPK/JNK activity after EtOH treatment in the acute (1-hour exposure) setting.

The observed anti-inflammatory effects of alcohol are in line with other studies reporting immunosuppressive properties of alcohol exposure in different models of inflammation [19, 20, 30]. Karavitis et al. have shown that in mice, a single binge of alcohol resulted in significantly decreased release of IL-6 and TNF-*α* from alveolar macrophages following LPS stimulation [38]. Jonsson and Palmblad described LPS-induced neutrophil adhesiveness to human umbilical vein endothelial cells, which physiologically coexpress E-selectin and IL-8 on their surface to induce the attachment signal for neutrophils, to be inhibited by acute (4 h long exposition) alcohol stimulation [39]. Our group also found comparable results when treating human lung epithelial cells with IL-1*β* and subsequently exposing them to alcohol (170 mM) for one hour [21]. In regard to dose dependency, MacGregor et al. found acute alcohol exposure of up to 1000 mg/dl to diminish neutrophil adhesion to endothelial monolayers in a dose-dependent manner [40]. In the underlying study, decreased neutrophil adhesion to stimulated HepG2 cells upon alcohol exposure was observed. However, no dose or time dependency occurred. The same results were raised when exposing Chang human liver cells to IL-1*β* and subsequently treating them with EtOH in the same dose and duration time. However, in the case of Chang liver cells, IL-6 release upon IL-1*β* stimulation and alcohol treatment was dose- as well as time-dependent, with only acute and high dose of alcohol to result in significantly diminished IL-6 release (own unpublished data). Summarized, this data might indicate that alcohol exerts its anti-inflammatory effects on different dose as well as time windows in different cell types and in corresponding cell lines.

This finding might be in line with Szabo et al., where it was proven that moderate alcohol consumption inhibits proinflammatory gene transcription through NF-*κ*B in monocytes, while promoting the production of anti-inflammatory cytokine IL-10 [41]. However, in contradiction, some studies suggest the opposite effect—Gavala et al. reported decreased cytokine production (including IL-10 and TNF-*α*) in dose-dependent alcohol exposure [42]. Taken altogether, all those findings and conclusions may indicate that alcohol is able to promote or suppress the proinflammatory responses depending probably on the conditions and setting (e.g., LPS stimulation); however, further investigation is still necessary.

The possible involved cellular signalling pathways indicate the involvement of the MAPK-STAT3 axis. Acute exposure of HepG2 to low-dose alcohol significantly reduced activation of SAPK/JNK, as well as STAT3, whereas either high dose or prolonged exposure to alcohol did not markedly modulate the phosphorylation and therefore activation of both regulatory proteins. This data is in line with the findings of Oak et al. who found enhanced JNK phosphorylation in LPS-stimulated human monocytes after exposure to 25 mM alcohol [43]. However, results contradictory to our data were acquired from another study. Chen et al. found acute exposure to alcohol to reduce IL-6-induced STAT3 activation in cultured HepG2; however, the group noticed the inhibition of this factor by EtOH in the primary hepatocytes isolated from rat models [44]. Interestingly, although we found a high dose of alcohol as well as prolonged exposure to show the same anti-inflammatory effects as acute exposure to a low dose of alcohol, no decrease in either SAPK/JNK or STAT3 activation after acute exposure to low-dose treatment has been observed. This may imply that alcohol exerts its effects *via* several cellular pathways in parallel. Additionally, alcohol immunosuppressive effects on HepG2 cells exerted *via* the MAPK-STAT3 axis might be sustained until the 72-hour endpoint, although activation of the regulating proteins cannot be observed anymore. However, further investigation in this matter is required, as our study is limited only to the activity of the pathway and not its regulatory mechanistic backbone in detail.

Summing up, we found a decrease of proinflammatory cytokine release from inflamed human liver cells upon their acute as well as prolonged exposure to alcohol. Similarly, neutrophil adhesion to stimulated HepG2 cells was significantly diminished by alcohol. However, these effects were not found to be dependent on either the dose or the duration of alcohol exposure. Therefore, our study confirms that alcohol is an immunomodulatory agent in inflamed human hepatocytes *in vitro.* When looking at possible underlying mechanisms, acute exposure to low-dose alcohol significantly downregulates the SAPK/JNK and STAT3 activation. These findings imply that alcohol exerts anti-inflammatory effects by inhibiting the MAPK-STAT3 axis in human liver cells *in vitro.* Other optional signalling pathways as discussed above have to be evaluated in further studies.

A limitation of our study is that here only two different doses as well as only two different incubation periods for alcohol exposure were applied. In order to gain further insights into the dose- and time-dependent effects of alcohol exposure, additional studies are warranted. This may be important to define the window, in which alcohol exerts its immune-modulatory and thereby potentially beneficial effects on cellular immunity. Additionally, other pro- and anti-inflammatory cytokines as well as signalling pathways should be analysed in further studies to gain more detailed insights into the immunomodulatory properties of alcohol. Furthermore, there are other promising approaches in terms of research of the JNK role in alcohol drinking. Yang et al. showed an interesting insight into the development of alcohol-induced liver steatosis and the involvement of cytochrome P4502E1 and JNK activation [45], while Yan et al. underlined the possible role of JNK in the binge drinking-related progression of atrial arrhythmia in the holiday heart syndrome [46]. Further research on the JNK role in chronic inflammatory diseases should be considered.

## Data Availability

Data are available upon a reasonable request to the corresponding author BR.
